# GADD45β induction by S-adenosylmethionine inhibits hepatocellular carcinoma cell proliferation during acute ischemia-hypoxia

**DOI:** 10.18632/oncotarget.9295

**Published:** 2016-05-11

**Authors:** Ding Ma, Baiyong Shen, Varun Seewoo, Hui Tong, Weiping Yang, Xi Cheng, Zhijian Jin, Chenghong Peng, Weihua Qiu

**Affiliations:** ^1^ Department of Surgery, Ruijin Hospital, Shanghai Jiao Tong University School of Medicine, Shanghai, China; ^2^ Department of Surgery, Huadong Hospital, Shanghai, China

**Keywords:** hepatocellular carcinoma, S-adenosylmethionine, ischemia-hypoxia, GADD45β, apoptosis

## Abstract

Growth arrest DNA damage-inducible gene 45β (GADD45β), which influences cell growth, apoptosis and cellular response to DNA damage, is downregulated in hepatocellular carcinoma (HCC). S-adenosylmethionine (SAMe) serves as an essential methyl donor in multiple metabolic pathways and is a polyamine and glutathione (GSH) precursor. In this study, we assessed the roles of GADD45β and SAMe in cell survival during acute ischemia-hypoxia (I/H). SAMe treatment induced growth of HL-7702 normal hepatic cells, but decreased the viability of HepG2 (*p53* wild-type) and Hep3B (*p53* null) HCC cells. Cells were exposed to I/H with or without SAMe pre-treatment. I/H exposure alone triggered HCC cell proliferation promoted by autophagy. SAMe pre-treatment restored GADD45β expression and activated HCC cell apoptosis and eliminated I/H-induced HCC cell proliferation. *p53* loss blunted the response to SAMe and I/H exposure in Hep3B cells; thus, the inhibitory effect of SAMe on cell proliferation may be reduced in *p53*-null cells as compared to wild-type cells. These results indicate that GADD45β induction by SAMe inhibits HCC cell proliferation during I/H as a result of increased apoptosis, and that SAMe also protects normal hepatocytes from apoptotic cell death and promotes normal cell regeneration. SAMe should be considered a potential therapeutic agent for the management of HCC.

## INTRODUCTION

Despite recent progress in diagnostic and therapeutic management, hepatocellular carcinoma (HCC) patient prognosis remains poor [1–4]. The level of downregulation of growth arrest DNA damage-inducible gene 45β (GADD45b) in HCC is strongly correlated with increased tumor malignancy [5]. GADD45β is associated with cell growth control, apoptosis and cellular responses to DNA damage [6]. Consequently, lack of GADD45β expression in HCC might lead to atypical cell growth or apoptosis. Therefore, GADD45b has been identified as a potential molecular marker and therapeutic target in both HCC and chronic liver diseases.

A characteristic of locally advanced solid tumors like HCC is tumor hypoxia, resulting from an imbalance in oxygen supply and consumption [7]. Our previous study showed that acute ischemia-hypoxia (I/H) exposure may trigger compensatory HCC cell proliferation, and that autophagy plays an important role in HCC cell survival during acute I/H [8, 9].

S-adenosylmethionine (SAMe) is an essential compound in many metabolic pathways, serving as a methyl donor and a precursor for polyamines and glutathione (GSH) [10]. Most patients with liver injury or a chronic liver disease, like cirrhosis, have impaired SAMe biosynthesis due to reduced MAT1A expression and MAT I/III inactivation, which are respectively the enzyme and isoenzyme involved in SAMe synthesis in normal hepatocytes [11, 12]. SAMe may inhibit tumor growth, reduce tumor invasiveness and slow metastasis through methyl group donation leading to gene hypermethylation and reduction of overall hypomethylation to inhibit oncogene expression [13–15].

In the present study, the effect of SAMe on cultured HepG2 (*p53*-wild type), Hep3B (*p53*-null) hepatoma cells and HL-7702 normal hepatic cells was investigated. On establishment of an acute I/H model *in vitro*, the impact of SAMe pre-treatment during I/H was studied, and the potential role of *p53* in the model was explored.

## RESULTS

### Effects of SAMe on cell viability

**Figure 1:**
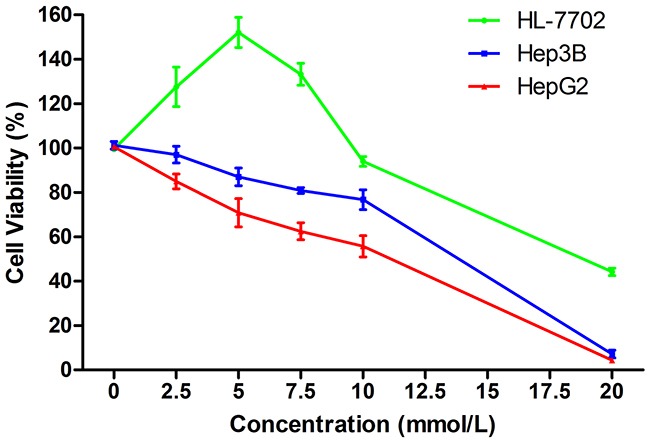
Effects of SAMe on cell viability Treatment with low SAMe concentrations (0-10 mmol/L) for 24 h induced HL-7702 cell growth, with an optimal increase in viability at 5 mmol/L. Higher concentrations (10-20 mmol/L) inhibited HL-7702 cell growth. SAMe treatment resulted in a dose-dependent inhibition of HepG2 and Hep3B cell proliferation.

The effects of SAMe on HCC and normal liver cell viability were examined. Exposure to low SAMe concentrations (0–10 mmol/L) for 24 h significantly induced HL-7702 cell growth. At the optimal concentration of 5 mmol/L, cell proliferation was enhanced by about 52% (152.09±4.78%). However, higher SAMe concentrations (10–20 mmol/L) and longer exposure times (48 or 72 h) inhibited HL-7702 growth. Meanwhile, SAMe treatment resulted in dose and time dependent inhibition of HepG2 and Hep3B cell proliferation. With 5 mmol/L SAMe for 24 h, HepG2 cell viability was reduced by about 29.2% (70.8±4.5%) and that of Hep3B by about 13.0% (87.1±2.8%). At SAMe concentrations that inhibited HL-7702 cell growth, cell viability was still higher than that of HepG2 and Hep3B. Therefore, in subsequent experiments cells were exposed to 5 mmol/L SAMe for 24 h; this concentration had the maximal pro-proliferation effect on HL-7702 cells, while the two hepatoma cell lines were adequately inhibited (Figure 1).

### Effects of acute I/H on cell proliferation with/without SAMe pre-treatment

**Figure 2:**
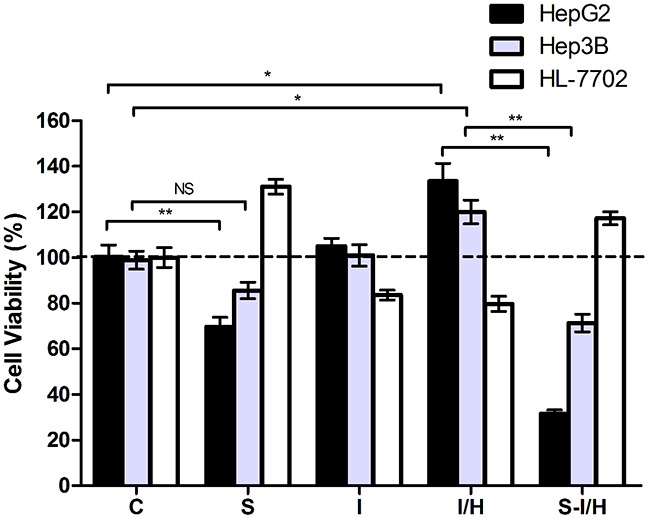
Effects of acute I/H on cell proliferation with/without SAMe pre-treatment Comparison of cell proliferation among Control (C), SAMe treatment (S), Ischemia (I), Ischemia-Hypoxia (I/H) and SAMe pre-treatment (S-I/H) groups. NS *p*>0.05; *p<0.05; **p<0.01

To explore the influence of SAMe on HCC during I/H, we established an I/H model as per our previous study [9]. Cell proliferation rates were examined across the different groups. I/H increased HepG2 proliferation by 33.2±7.8% (*p*<0.05) (Figure 2). Meanwhile, SAMe inhibited HepG2 cell growth by about 30.6±4.2% (*p*<0.05). The SAMe inhibitory effect was further enhanced when pre-treated HepG2 cells were exposed to I/H. Cell viability of group S-I/H was only 31.6±1.6% (group I/H: 133.4±7.8% *vs.* group S-I/H: 31.6±1.6%, *p<*0.05).

A comparable effect was observed in Hep3B cells. Cell viability was slightly reduced by 13.2±3.6% after SAMe treatment (group S: 85.6±3.6% *vs.* group C: 98.8±3.7%, *p*>0.05), but after I/H exposure, cell viability increased by 20.1±5.2%. The SAMe inhibitory effect was again enhanced when pre-treated cells were exposed to I/H, with a resulting cell viability of 71.2±3.8% (group I/H: 119.9±5.2% *vs.* group S-I/H: 71.2±3.8%, *p<*0.05).

HL-7702 cell proliferation was markedly induced 31.0±3.2% by SAMe, while ischemia and I/H led to 16.4±2.2% and 20.3±3.4% decreases in cell viability, respectively. A 17.2±2.9% proliferation increase was measured with SAMe pre-treatment by followed by I/H.

We further explored the effects of acute I/H on HCC cells with different *p53* contexts (*p53* wild-type and null). To eliminate differences in growth characteristics between HepG2 and Hep3B cells, proliferation rates were compared according to SAMe pre-treatment and/or I/H exposure. The SAMe inhibitory effect on Hep3B cell proliferation was reduced compared to HepG2 cells (13.2±3.6% *vs.* 30.6±4.2%, *p<*0.05) (Figure 2). Exposure to acute I/H induced HepG2 and Hep3B cell proliferation with no significant difference between the two lines (33.2±7.8% *vs.* 21.1±5.2%, *p*>0.05). When SAMe pre-treatment was followed by I/H exposure, Hep3B cell proliferation was increased as compared to HepG2 cells (71.2±3.8% *vs.* 31.6±1.6%, *p<*0.01). Furthermore, HepG2 cell proliferation was increased by SAMe pre-treatment alone as compared to pre-treatment followed by I/H (69.6±4.2% *vs.* 31.6±1.6%, *p<*0.01); this difference was not observed in Hep3B cells (85.6±3.6% *vs.* 71.2±3.8%, *p*>0.05).

These results showed that I/H increased HCC cell viability. In addition to inhibiting HCC cell proliferation alone, SAMe pre-treatment reversed I/H-induced cell proliferation. Contrary growth patterns were observed in human normal hepatocyte HL-7702 cells; I/H reduced proliferation while SAMe promoted it. Pre-treatment with SAMe allowed normal hepatocytes to survive I/H. Consistent with our previous study [9], *p53* might play an important role in the effect of SAMe on HCC cells.

### Acidic vesicular organelles (AVOs) in cells during I/H, with/without SAMe pre-treatment

**Figure 3:**
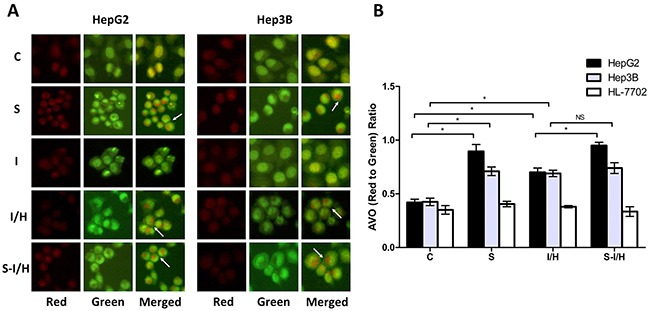
AVOs in cells during I/H, with/without SAMe pre-treatment Qualitative AVO analyses **A.** Staining of live HepG2 and Hep3B cells for AVOs revealed the formation of LC3 puncta. Quantitative AVO analyses **B.** AVOs were quantitatively assessed according to the red-to-green fluorescence ratio obtained using Photoshop software. NS *p*>0.05; *p<0.05.

Our previous study showed that acute I/H exposure may trigger compensatory HCC cell proliferation, and that autophagy plays an important role in HCC cell survival during acute I/H [9]. Autophagy is characterized by AVO formation, and acridine orange staining was used to morphologically detect AVOs. Typical acridine orange accumulation in acidic AVOs appears as granular bright red fluorescence in the cytoplasm, indicating autophagosome formation. Acridine orange staining of live HepG2 and Hep3B cells showed increased AVO formation following SAMe pre-treatment (Figure 3A). Consistent with our previous results [4], I/H exposure increased AVOs amount in both HepG2 and Hep3B cells. Acute I/H with SAMe pre-treatment had no apparent influence on AVO formation in Hep3B cells, while a notable increase in AVOs was observed in HepG2 cells. AVO accumulation in HL-7702 cell cytoplasm was not changed by SAMe and/or I/H treatment.

The red-to-green fluorescence ratio (AVO accumulation) increased compared to controls in both HepG2 and Hep3B cells after I/H exposure (HepG2: 0.70±0.04 *vs.* 0.42±0.03, *p<*0.05; Hep3B: 0.69±0.03 *vs.* 0.43±0.04, *p<*0.05) (Figure 3B). Additionally, the red-to-green fluorescence ratio increased in SAMe-treated HepG2 and Hep3B cells (group S) compared to controls (HepG2: 0.90±0.07 *vs.* 0.42±0.03, *p<*0.05; Hep3B: 0.71±0.04 *vs.* 0.43±0.04, *p<*0.05), suggesting that SAMe treatment could induce autophagy. When HepG2 cells were pre-treated with SAMe and then exposed to I/H (group S-I/H), AVO formation increased significantly (groups S-I/H *vs*. I/H: 0.95±0.03 *vs.* 0.70±0.04, *p<*0.05). SAMe pre-treatment failed to enhance I/H exposure-induced autophagy in Hep3B cells (groups S-IH *vs*. I/H: 0.74±0.05 *vs.* 0.69±0.03, *p*>0.05). While a mild increase in autophagosome formation was observed in HL-7702 cell I/H and S groups, no changes were statistically significant.

These data showed that SAMe and/or acute I/H exposure induced autophagy in HepG2 and Hep3B cells, but not HL-7702 cells. SAMe pre-treatment triggered autophagy only in HepG2 cells.

### Beclin 1 expression during I/H, with/without SAMe pre-treatment

**Figure 4:**
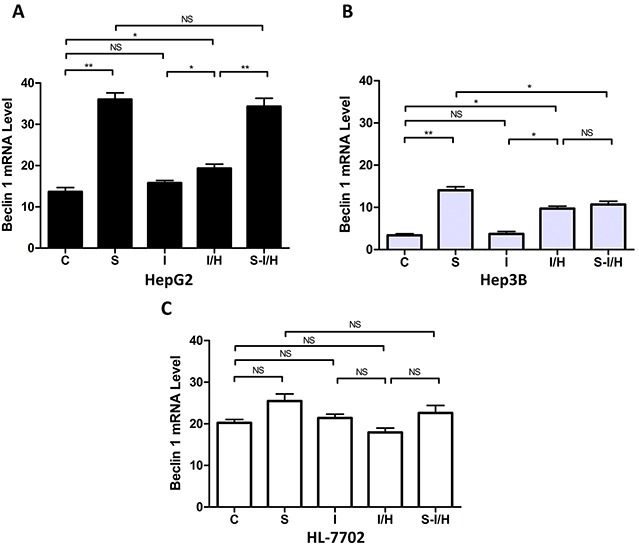
Beclin 1 expression during I/H, with/without SAMe pre-treatment Beclin 1 levels in HepG2 (*p53* wild type) **A.** Hep3B (*p53* null) **B.** and HL-7702 (normal liver) **C.** cells in all treatment groups. NS *p*>0.05; *p<0.05; **p<0.01.

Beclin 1 mRNA levels were evaluated to confirm autophagy activation following SAMe and/or acute I/H exposure [16]. Beclin 1 expression was increased in SAMe-treated HepG2 cells (group C: 13.4±1.2 *vs*. group S: 37.2±1.8, *p<*0.01) (Figure 4A). Only a minimal increase in autophagy was observed with ischemia (group I: 15.8±1.0 *vs.* group C: 13.4±1.2, *p*>0.05). In contrast, I/H induced autophagy in HepG2 cells, and Beclin 1 levels increased from 13.4±1.2 in group C to 21.9±0.7 in group I/H (*p<*0.05). When HepG2 cells were treated with SAMe prior to I/H (group S-I/H), there was little change in Beclin 1 expression compared with group S, but there was a significant increase as compared with group I/H (S-I/H: 34.7±1.6 *vs.* S: 37.2±1.8, *p*>0.05; S-I/H: 34.7±1.6 *vs.* I/H: 21.9±0.7, *p<0.01*).

Similar autophagic activity changes occurred in Hep3B cells (Figure 4B). After SAMe treatment, Beclin 1 expression increased from 3.4±0.4 in controls to 14.1±0.8 in group S (*p<0.01*). Ischemia alone (group I) barely induced autophagy and Beclin 1 levels were unchanged (3.7±0.6 *vs*. 3.4±0.4 in controls, *p*>0.05). However, I/H triggered a Beclin 1 expression increase to 9.7±0.6 (group I/H, *p<*0.05). Pre-treatment with SAMe did not further enhance Beclin 1 levels (group I/H: 9.7±0.6 *vs.* group S-I/H: 10.7±0.8, *p*>0.05).

Similar to the results of the AVO formation assay, Beclin 1 expression in HL-7702 cells was unchanged by the treatments. Beclin 1 levels marginally increased with SAMe treatment, decreased after I/H and were slightly restored by SAMe pre-treatment before I/H (C: 20.2±0.8, S: 25.5±1.7, I/H: 18.0±1.0, S-I/H: 22.6±1.8; groups C *vs.* S, *p*>0.05; groups C *vs.* I/H, *p*>0.05; groups S *vs.* S-I/H, *p*>0.05; groups I/H *vs.* S-I/H, *p*>0.05) (Figure 4C). Steady state Beclin1 levels in hepatoma cell lines HepG2 and Hep3B were significantly lower than that in normal hepatocyte HL-7702 cells, with levels in HepG2 lower than those in Hep3B cells. Beclin 1 expression in HepG2, Hep3B and HL-7702 cells was 13.4±1.2, 3.4±0.4 and 20.2±0.8, respectively (HepG2 *vs.* HL-7702, *p<*0.05; Hep3B *vs.* HL-7702, *p<*0.01).

Consistent with our previous study [9], I/H and SAMe treatment both induced autophagy in HCC cells. Furthermore, Beclin 1 expression differences between the two HCC cell lines suggested that *p53* might play a role in autophagy regulation in response to I/H after SAMe pre-treatment.

### Effects of autophagy on HCC cell proliferation during acute I/H, with/without SAMe pre-treatment

An essential autophagy gene, ATG7 is an E1-like enzyme involved in MAP-LC3 ubiquitination early in the pathway, and ATG7 knockdown inhibits autophagy specifically [17]. HCC cells were transfected with ATG7 small interfering RNAs (siRNAs) to assess the effects of autophagy during acute I/H with or without SAMe pre-treatment.

**Figure 5:**
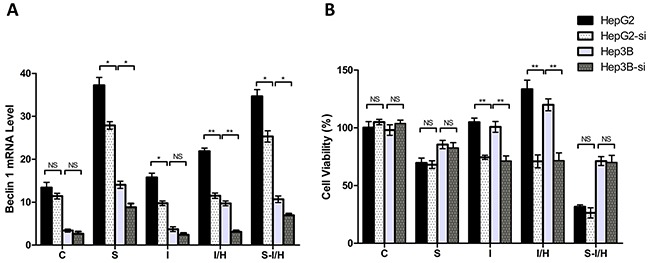
Effects of autophagy on HCC cell proliferation during acute I/H with/without SAMe pre-treatment Beclin 1 levels **A.** and cell proliferation rates **B.** in all groups after autophagy inhibition via ATG7 siRNA, as compared to un-transfected cells. HepG2-si: ATG7 siRNA interference in HpeG2 cells. Hep3B-si: ATG7 siRNA interference in Hep3B cells. NS *p*>0.05; *p<0.05; **p<0.01.

ATG7 interference inhibited Beclin 1 increases induced by I/H treatment in both HepG2 and Hep3B cells (Figure 5A). Beclin 1 levels in HepG2 and Hep3B cells decreased approximately 47.4% and 68.4%, respectively, during I/H following ATG7 knockdown (group I/H-si *vs.* I/H, HepG2: 11.5±0.6 *vs.* 21.9±0.7, Hep3B: 3.1±0.3 *vs.* 9.7±0.6, *p<*0.01). A comparable effect was observed in group S-si as well. ATG7 knockdown inhibited SAMe-induced Beclin 1 expression in HepG2 and Hep3B cells. Beclin 1 levels decreased from 37.2±1.8 to 27.9±0.9 in HepG2 (*p<*0.05) and from 14.1±0.8 to 8.8±0.9 in Hep3B cells (*p<*0.05). Similar to our previous results, ATG7 knockdown inhibited Beclin 1 expression in HepG2 and Hep3B cells in group S-I/H-si compared with group S-I/H (HepG2: 25.3±1.3 *vs.* 34.7±1.6, *p<*0.05, Hep3B: 6.9±0.4 *vs.* 10.7±0.8, *p<*0.05). These results showed that ATG7 knockdown inhibited autophagy in almost all treatment groups.

ATG7 knockdown alone had almost no inhibitory effect on HepG2 and Hep3B cell proliferation (group C *vs.* C-si, HepG2: 100.3±5.2% *vs.* 105.0±2.3%, *p*>0.05; Hep3B: 98.1±4.6% *vs.* 103.8±2.9%, *p*>0.05) (Figure 5B). AtG7 knockdown inhibited I/H-induced proliferation in HepG2 and Hep3B cells approximately 46.8% and 40.3%, respectively (group I/H *vs.* I/H-si, HepG2: 133.4±7.8% *vs.* 71.0±5.6%, Hep3B: 119.9±5.2% *vs.* 71.6±6.7%, *p<*0.01). Meanwhile, ATG7 knockdown only minimally influenced proliferation in groups S-si and S-I/H-si in both HepG2 and Hep3B cells (group S *vs.* S-si, HepG2: 69.6±4.2% *vs.* 68.1±3.3%, Hep3B: 85.6±3.6% *vs.* 82.6±4.5%, *p*>0.05; group S-I/H *vs.* S-I/H-si, HepG2: 31.6±1.6% *vs.* 26.4±4.4%, Hep3B: 71.2±3.8% *vs.* 70.0±6.2%, *p*>0.05).

These results collectively suggested that the observed inhibitory effect of ATG7 knockdown on HCC cell proliferation following acute I/H exposure most likely resulted from ATG7 knockdown-induced autophagy inhibition. Importantly, ATG7 knockdown did not influence HCC cell proliferation during acute I/H with SAMe pre-treatment. These results indicated that in addition to autophagy, other mechanisms may be involved in elimination of I/H-induced HCC cell proliferation by SAMe pre-treatment.

### Apoptotic response of HCC cells during acute I/H with/without SAMe

**Figure 6:**
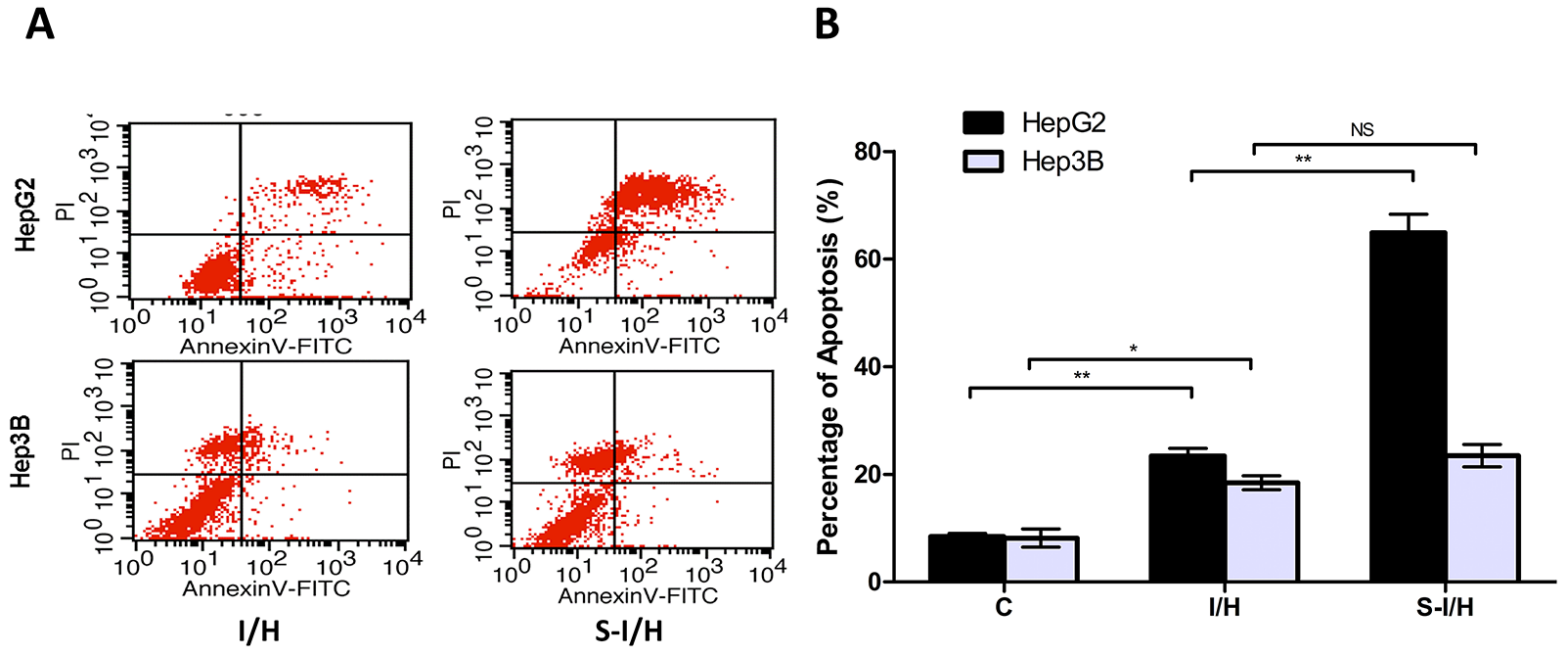
HCC cell apoptosis during acute I/H, with/without SAMe pre-treatment HepG2 and Hep3B cell apoptosis was investigated using Annexin V-FITC and PI. (AnV^+^) PI^−^ cells were considered early apoptotic and (AnV^+^) PI^+^ cells were considered late apoptotic and necrotic. NS *p*>0.05; *p<0.05; **p<0.01.

We performed flow cytometry to assess the apoptotic response of HCC cells to SAMe treatment. The percentage of apoptotic cells following I/H exposure increased from 8.5±0.6% to 23.4±1.4% (*p<*0.01) in HepG2 and from 8.2±1.7% to 18.4±1.3% (*p<*0.05) in Hep3B cells (Figure 6). When first pre-treated with SAMe and then exposed to I/H, the total fraction of apoptotic HepG2 cells increased to 64.9±3.4% (*p<*0.01); no change was observed in Hep3B cells (23.5±2.1%, *p*>0.05). The apoptotic effect mostly resulted from an increased percentage of cells in late apoptosis (AnnexinV^+^/PI^+^). Therefore, the inhibitory effect of SAMe on I/H-induced HCC cell proliferation was accomplished via activation of the apoptotic pathway.

### Influence of I/H on GADD45β expression with/without SAMe pre-treatment

**Figure 7:**
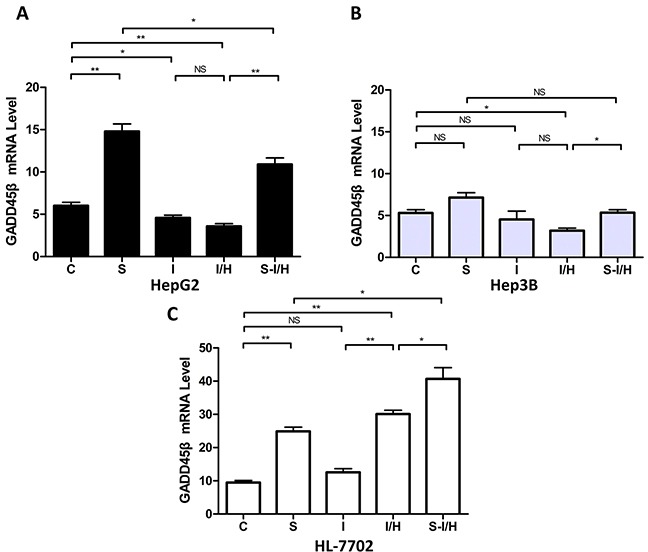
Influence of I/H on GADD45β expression with/without SAMe pre-treatment **(A)** GADD45β levels in HepG2 **A.** Hep3B **B.** and HL-7702 **C.** cells in all groups.NS *p*>0.05; *p<0.05; **p<0.01.

Our previous study showed that SAMe induced GADD45β via NF-κB [18] and NF-κB played an important role in cell apoptosis. Here, the influence of I/H on GADD45β expression with and without SAMe was examined. When HepG2 cells were exposed to 5 mmol/L SAMe for 24 h, GADD45β expression was 14.8±0.9 compared with 6.0±0.4 in the control group (*p<*0.01) (Figure 7A). Moreover, both ischemia and I/H inhibited GADD45β expression (decreased from 6.0±0.4 to 4.6±0.3 in group I, *p<*0.05; and to 3.6±0.3 in group I/H, *p<*0.01). GADD45β expression between groups I and I/H was unchanged (*p*>0.05). Pre-treatment with SAMe (group S-I/H) restored GADD45β expression inhibited by I/H (increased to 10.9±0.8, *p<*0.01).

Also shown in our previous study [18], SAMe induced GADD45β in Hep3B cells. GADD45β expression increased from 5.3±0.4 in controls (group C) to 7.1±0.6 in group S (*p*>0.05). However, in HepG2 cells, only I/H decreased GADD45β expression (to 3.2±0.3) compared with controls (*p<*0.05). Moreover, SAMe pre-treatment barely increased GADD45β expression during I/H to 5.3±0.4 (S-I/H *vs.* I/H *p<*0.05, Figure 7B).

SAMe treatment in HL-7702 cells (Figure 7C) upregulated GADD45β; expression increasing to 24.9±1.2 in group S compared with 9.5±0.6 in the control group (*p<*0.01). Ischemia alone (group I) only mildly increased GADD45β levels, to 12.6±1.1 compared with 9.5±0.6 in the control group (*p*>0.05). However, I/H increased GADD45β levels to 30.1±1.2 from control group levels (*p<*0.01), and pre-treatment with SAMe (group S-I/H) further enhanced GADD45β expression during I/H. GADD45β expression was 40.7±3.4 in group S-I/H (S-I/H *vs.* I/H, *p<*0.05).

Further analysis showed that basal GADD45β levels in HepG2 and Hep3B cells were lower compared with HL-7702 cells. GADD45β expression was 6.0±0.4, 5.3±0.4 and 9.5±0.6 in HepG2, Hep3B and HL-7702 cells, respectively. GADD45β expression in the two HCC cell lines was not statistically different.

As an important downstream regulator of *p53*, GADD45β status also regulated apoptosis activation on I/H treatment. Our results demonstrated that SAMe increased GADD45β expression in Hep3B cells to a greater extend than in HepG2 cells, suggesting that *p53* loss might be responsible for the failure of SAMe to induce GADD45β expression. Similar results were observed in terms of apoptosis activation.

Together, our results suggested that during the process of acute I/H, SAMe increased GADD45β expression and consequently activated apoptosis in HCC cells to suppress tumor proliferation. *p53* status also appeared to influenced apoptotic activity.

## DISCUSSION

Elevated HCC interstitial pressure and high cellular proliferation rates lead to different kinds of I/H and reperfusion, which are key components of many pathological conditions [17, 19, 20]. Although transcatheter arterial chemoembolization (TACE) has significantly improved survival in HCC patients, I/H resulting from TACE may allow surviving cancer cells to proliferate and metastasize, resulting in tumor recurrence [21, 22]. In this study, we confirmed that acute I/H could enhance proliferation in the HepG2 and Hep3B hepatoma cell lines, and cellular compensation for I/H complicated HCC growth, resistance, recurrence and metastasis [23].

Our previous studies showed that SAMe can restore GADD45b expression in HCC and consequently inhibit HCC cell growth, invasiveness and metastasis [24]. In this study, low concentrations of SAMe induced growth of HL-7702 cells, but decreased HepG2 and Hep3B cell viability, which revealed the dual roles of SAMe in HCC cell inhibition and normal liver cell maintenance. Because the response of HCC cells to SAMe treatment during acute I/H was largely unknown, the effects of acute I/H on cell proliferation with and without SAMe pre-treatment were measured. Results showed that HCC cell proliferation resulting from I/H was largely inhibited by SAMe pre-treatment. Importantly, inhibition of HCC cell proliferation by SAMe pre-treatment with I/H exposure was more apparent than with SAMe treatment alone. The opposite results were observed in normal liver cells.

Metabolic stress, including nutrient deprivation, growth factor deprivation and hypoxia can induce autophagy [25, 26]. In this study, as evidenced by qualitative and quantitative Beclin 1 detection, we confirmed that autophagy in HCC cells increased on exposure to I/H. Autophagy provides a temporary source of metabolic energy and catabolism intermediates [27] and therefore enables cell survival in nutrient-limited environments [28]. However, the role of autophagy in cancer is still debated [29]. Autophagy may enhance or suppress tumorigenesis. Some autophagy-inducing therapeutic strategies, like Rapamycin, have benefited patients [30]. Similarly, our results illustrated that SAMe, while inhibiting HepG2 and Hep3B cell proliferation, also activated autophagy. Beclin 1 expression increased dramatically in both HepG2 and Hep3B cells with SAMe treatment. HepG2 cell autophagy was further activated with SAMe pre-treatment followed by I/H exposure, but this was not observed in Hep3B cells. Autophagy inhibition via ATG7 knockdown inhibited the increase in Beclin 1 expression induced by SAMe, I/H treatment, and SAMe pre-treatment in both HepG2 and Hep3B cells. However, ATG7 knockdown had only minor inhibitory effects on HCC cell proliferation in group S-I/H, while proliferation in group I/H was largely inhibited. These results indicated that autophagy was not the only mechanism by which SAMe pre-treatment eliminated I/H-induced cell proliferation. SAMe induced both autophagy and apoptosis, but whether autophagy was an active death-inducing mechanism (cell death by autophagy) or the result of an unsuccessful effort to prolong survival of damaged cells (cell death with autophagy) was difficult to distinguish. Flow cytometry results showed that the percentage of apoptotic HCC cells exposed to I/H alone was much less than in cells pre-treated with SAMe. Therefore, our results suggest that inhibition of HCC cell proliferation via SAMe treatment is accomplished by activating apoptosis.

As a positive apoptosis modulator, GADD45β activation after exposure to genotoxins prevents propagation of damaged cells, causing cell growth arrest and subsequent apoptosis [31]. GADD45β induction could inhibit HCC cell growth and trigger apoptosis via the NF-κB pathway [17]. In this study, SAMe treatment induced GADD45β expression in HCC cells, but I/H exposure only barely did so. Only with SAMe pre-treatment could GADD45β be induced by I/H stimulation. On the contrary, GADD45β expression in normal liver cells was sensitive to both SAMe and I/H treatment, and SAMe pre-treatment further activated GADD45β expression already upregulated by I/H. The blunt response of HCC cells to hypoxia exposure indicated that low GADD45β expression in HCC might lead to a failure to inhibit atypical cell growth or to trigger apoptosis. We hypothesized that autophagy might be responsible for promoting HCC cell proliferation during I/H, and induction of GADD45β expression by SAMe pre-treatment was key to inhibiting HCC cell proliferation under I/H conditions via apoptosis.

The GADD45 gene family members are reportedly downstream effectors of *p53* required for cell cycle arrest following DNA damage [32], and *p53* mutation is closely associated with GADD45β downregulation in HCC. The distinct difference in *p53* status between HepG2 (*p53*-wild type) and Hep3B (*p53*-null) cells makes them a useful model with which to investigate the role of *p53* in SAMe-mediated responses to I/H. *p53*-null cells were reportedly insensitive to metabolic stress with a higher intracellular ATP level, but our results demonstrated that autophagy in Hep3B cells was stimulated by I/H. However, SAMe pre-treatment followed by I/H further activated autophagy in HepG2, but not Hep3B cells, showing that *p53* status influenced autophagy activation. Although *p53* loss might be responsible for limited SAMe-induced GADD45β expression in Hep3B cells, apoptosis was still partially activated by SAMe pre-treatment followed by I/H. Thus, SAMe-induced proliferation inhibition may be reduced in *p53*-null cells compared to wild-type cells. Our results related to *p53* status confirmed that autophagy may be responsible for promoting cell proliferation during I/H, and that apoptosis plays a main role in SAMe-induced proliferation inhibition.

In conclusion, GADD45β induction by SAMe enhanced its inhibitory effects on cancer cell proliferation during I/H by increasing apoptosis. SAMe protected normal hepatocytes against apoptotic cell death and promoted normal cell regeneration. Consequently, SAMe should be considered a potential therapeutic agent in the management of HCC.

## MATERIALS AND METHODS

### Cell culture, I/H simulation and SAMe treatment

The human hepatoma cell lines HepG2 and Hep3B were purchased from ATCC, while the established immortalized human fetal hepatic cell line, HL-7702, was purchased from China Center for Type Culture Collection (CCTCC, Wuhan, China) [33]. Cells were cultured as described previously [5].

Acute I/H models were established as described previously [9]. Briefly, ischemia was introduced via ischemia mimetic solution (glucose deprivation solution) (pH 6.6, 125 mmol/L NaCl, 8 mmol/L KCl, 1.2 mmol/L KH_2_PO_4_, 1.25 mmol/L MgSO_4_, 1.2 mmol/L CaCl_2_, 6.25 mmol/L NaHCO_3_, 5 mmol/L Sodium Lactate, and 20 mmol/L HEPES) [34]. Cells were placed in a hypoxic chamber equilibrated with 95% N_2_, 4% CO_2_, and 1% O_2_ for two hours (ischemia-hypoxia, group I/H). Glucose deprivation *in vitro* was utilized to reduce hypoxic response inhibition [35]. Cells treated with ischemia mimetic solution and exposed to normoxia were included as a control for hypoxia (ischemia-normoxia, group I). To assess the effect of SAMe on I/H, cells were pre-treated with 5 mmol/L SAMe (Knoll Farma- ceutici S.P.A., Milan, Italy) for 24 h and then I/H was introduced as described above (SAMe-ischemia-hypoxia, group S-I/H). Cells treated with 5 mmol/L SAMe for 24 h acted as negative controls (group S), while cells treated with culture media served as the system control (group C). Two hours after treatment, cells from all groups were harvested at corresponding time points in a parallel manner.

### Determination of SAMe dosages and cell proliferation

HepG2, Hep3B and HL-7702 cells were plated in triplicate at 5,000 cells/well in 100μL media in separate 96-well microtiter plates. The next day, 100μL SAMe was added to the wells at a final dosage of 0, 2.5, 5.0, 7.5, 10 or 20 mmol/L and plates were incubated for an additional 24 hours. Then, 20 μL of CCK 8 cell-counting solution (Dojindo Laboratories, Japan) was added to each well and incubated at 37°C for 3 hours. Absorbance was read spectrophotometrically at 450 nm with a reference at 650 nm using a microtiter plate reader (Tecan Safire 2). Cell viability was calculated according to the following formula: Cell proliferation (%) = [(A450sample-A450blank)/(A450control-A450blank)-1]*100. A dose-response curve was constructed to determine the concentration of SAMe that maximally induced HL-7702 proliferation and inhibited HepG2 and Hep3B cell growth.

### Beclin 1 and GADD45β mRNA detection by quantitative real-time PCR

Steady state Beclin 1 and GADD45β mRNA levels were determined by real-time PCR using SYBR Premix Ex Taq (TaKaRa, Japan). After treatment, total RNA was extracted and reversely transcribed to cDNA using Revert Aid First-strand cDNA Synthesis Kit (Fermentas, Canada). GAPDH was used as an endogenous control. Primers pairs were: Beclin 1: 5′-AGC TGC CGT TAT ACT GTT CTG-3′ and 5′-ACT GCC TCC TGT GTC TTC AAT CTT-3′; GADD45β: 5′-GGA CCC AGA CAG CGT GGT CCT CTG-3′ and 5′-GTG ACC AGG AGA CAA TGC AGG TCT-3′; GAPDH: 5′-GAA GGT GAA GGT CGG AGTC-3′ and 5′-GAA GAT GGT GAT GGG ATT TC-3′. Thermal cycling was performed in a 7000 Sequence Detection System (Applied Biosystems). PCR amplification conditions were as follows: 95°C for 10 min, 40 cycles of 95°C for 15 s, and 60°C for 60 s. Ct data was obtained automatically and each data point was performed in triplicate. Relative gene expression was calculated and expressed as 2 -ΔCt.

### AVO detection with acridine orange staining

After treatment, acridine orange (Sigma) was added to culture medium at a final concentration of 1 μg/mL followed by incubation in the dark at room temperature for 15 min. Samples were examined under a fluorescence microscope (Olympics IX71). AVO fluorescence was measured with an excitation wavelength of 488 nm and an emission wavelength of 515 nm. AVOs were quantitated according to the red-to-green fluorescence ratio obtained using Photoshop software (Adobe, San Jose, CA).

### Autophagy inhibition by RNA interference

ATG7 is essential for the autophagy conjugation system, autophagosome formation and starvation-induced degradation of proteins and organelles in mammalian cells [36]. To inhibit autophagy, ATG7 siRNA was transfected into HepG2, Hep3B and HL-7702 cells. The ATG7-targeting sense sequence and the Universal Negative Control siRNA were purchased from Invitrogen (12935-400, Invitrogen, Carlsbad, CA). The human ATG7 sequence (5′-GGAGTCACAGCTCTTCCTT-3′) was cloned into *Bam*H1 and *Eco*R1 sites of the pGSU6-GFP vector (GTP600300, Genlantis, San Diego, CA). siRNA plasmids were transfected using Lipofectamine 2000 (11668019, Invitrogen). Forty-eight hours after transfection, cells were collected by flow cytometry sorting. The irrelevant nucleotides did not targeting any annotated human genes were served as a negative control.

### Apoptosis assay

HepG2 and Hep3B cell apoptotic status after treatment was evaluated by measuring phosphatidylserine exposure on cell membranes using Annexin V-fluorescein isothiocyanate (Annexin V-FITC) and propidium iodide (PI) staining via the BD Pharmingen Annexin V-FITC Apoptosis Detection Kit I (BD Biosciences). After treatment, cell pellets were centrifuged and washed twice with cold PBS and suspended in 100 μl binding buffer. Cells were incubated with 5 μl Annexin V-FITC and 5 μl PI at room temperature for 15 min in the dark. An additional 400 μl of 1× binding buffer was added to each tube. Samples were analyzed immediately by flow cytometry (Becton Dickinson Facscalibur). 10000 events were acquired using green channel FL1 for Annexin V-FITC and the red channel FL3 for PI. (AnV^+^) PI^−^ cells were considered early apoptotic, and (AnV^+^) PI^+^ cells were considered late apoptotic and necrotic. Both subpopulations were counted together and expressed as the total fraction of apoptotic cells.

### Statistical analysis

Statistical analysis was performed using SPSS software (v.13.0). Data was expressed as mean ± SD of at least three independent experiments. Quantitative data were analyzed using Student's *t* test between two groups or by one-way ANOVA for multiple groups. A statistically significant difference was defined as *p*<0.05.
